# Calcium calmodulin kinase II activity is required for cartilage homeostasis in osteoarthritis

**DOI:** 10.1038/s41598-021-82067-w

**Published:** 2021-03-11

**Authors:** Giovanna Nalesso, Anne-Sophie Thorup, Suzanne Elizabeth Eldridge, Anna De Palma, Amanpreet Kaur, Kiran Peddireddi, Kevin Blighe, Sharmila Rana, Bryony Stott, Tonia Louise Vincent, Bethan Lynne Thomas, Jessica Bertrand, Joanna Sherwood, Antonella Fioravanti, Costantino Pitzalis, Francesco Dell’Accio

**Affiliations:** 1 Department of Veterinary Pre-Clinical Sciences, School of Veterinary Medicine, University of Surrey, Daphne Jackson Road, Guildford, GU2 7AL UK; 2 Barts and the London School of Medicine and Dentistry, William Harvey Research Institute, Queen Mary University of London, Charterhouse Square, London, EC1M 6BQ UK; 3 MRC Clinical Trials Unit, Institute of Clinical Trials and Methodology, UCL, London, UK; 4 Clinical Bioinformatics Research, London, UK; 5 Imperial College, London, UK; 6 Kennedy Institute of Rheumatology, University of Oxford, Oxford, UK; 7 Department of Orthopaedic Surgery, Otto-von-Guericke University, Magdeburg, Germany; 8 Institute of Musculoskeletal Medicine, University Hospital Münster, Münster, Germany; 9 Rheumatology Unit, Azienda Ospedaliera Universitaria Senese, Policlinico Le Scotte, Siena, Italy

**Keywords:** Cell signalling, Mechanisms of disease

## Abstract

WNT ligands can activate several signalling cascades of pivotal importance during development and regenerative processes. Their de-regulation has been associated with the onset of different diseases. Here we investigated the role of the WNT/Calcium Calmodulin Kinase II (CaMKII) pathway in osteoarthritis. We identified Heme Oxygenase I (HMOX1) and Sox-9 as specific markers of the WNT/CaMKII signalling in articular chondrocytes through a microarray analysis. We showed that the expression of the activated form of CaMKII, phospho-CaMKII, was increased in human and murine osteoarthritis and the expression of HMOX1 was accordingly reduced, demonstrating the activation of the pathway during disease progression. To elucidate its function, we administered the CaMKII inhibitor KN93 to mice in which osteoarthritis was induced by resection of the anterior horn of the medial meniscus and of the medial collateral ligament in the knee joint. Pharmacological blockade of CaMKII exacerbated cartilage damage and bone remodelling. Finally, we showed that CaMKII inhibition in articular chondrocytes upregulated the expression of matrix remodelling enzymes alone and in combination with Interleukin 1. These results suggest an important homeostatic role of the WNT/CaMKII signalling in osteoarthritis which could be exploited in the future for therapeutic purposes.

## Introduction

Osteoarthritis (OA) is a joint disease driven by pathological biomechanics and characterized by cartilage breakdown, subchondral bone sclerosis, osteophytosis, and inconstant low degree inflammation^1^. OA is a leading cause of chronic disability worldwide: patients suffer from joint pain and impaired motility, which reduces their independence^2^. No therapeutic treatment other than symptomatic relief is currently available, leaving joint replacement surgery as the ultimate option.

OA progression is driven by an imbalance between cartilage degradation and reparative, homeostatic responses. Pathological biomechanical stimuli activate signalling pathways including interleukin 1 beta (IL1B)^3^ and the WNT pathway^4^, which in turn upregulate matrix metalloproteases (MMPs) and A Disintegrin and Metalloproteinase with Thrombospondin motifs (ADAMTS) aggrecanases, which contribute to the degradation of the cartilage extracellular matrix^5^.

Excessive activation of the WNT signalling pathway has been linked to the onset and progression of cartilage degeneration in OA, both in human and in mice^6–9^. WNTs are a family of 19 highly conserved morphogens capable of activating multiple signalling cascades. The most characterised one is the WNT beta-catenin-dependent pathway. The WNT/beta-catenin pathway (also known as canonical WNT pathway) is activated by the interaction of WNT ligands with Frizzled (FZDs) and Lipoprotein-related Protein Receptor 5 and 6 (LRP5/6) which leads to the intracellular accumulation of beta-catenin and its translocation to the nucleus. Within the nucleus, beta-catenin interacts with T-cell factor/lymphoid enhancer-binding factor (TCF/LEF) transcription factors promoting the transcription of target genes such as *AXIN2*^10^. Other, so called “non-canonical” WNT pathways, are activated by the interaction of WNT ligands with FZDs and other co-receptors such as Receptor Tyrosine Kinase (RYK) and Retinoic Acid-Related Orphan Receptor (RORs), driving the activation of downstream signalling cascades activated by intracellular calcium release and/or associated with intercellular communication to coordinate polarity between adjacent cells^11^.

Ca^2+^Calmodulin-dependent Kinase II (CaMKII) is an intracellular kinase that can be activated by several stimuli including WNTs^12^ and inflammatory stimuli^13,14^. CaMKII is a Ser/Thr kinase expressed in mammals in 4 isoforms, alpha, beta, gamma and delta^15^. Binding of Ca^2+^/Calmodulin to CaMKII transiently activates it. Once activated, CaMKII phosphorylates its substrates, including itself. The autophosphorylation of CaMKII in T287 results in its constitutive activation, even in the absence of further stimulation^16^. WNT-induced CaMKII activity regulates chondrocyte differentiation^12^.

We showed that a single WNT ligand could simultaneously activate the beta-catenin-dependent and the CaMKII-dependent WNT pathways with distinct biological outcomes^12^.

These data suggest an important and still uncharacterised role for the WNT/CaMKII pathway in the maintenance of cartilage homeostasis. To test this hypothesis, the current study investigates the role of the WNT/CaMKII pathway in the context of osteoarthritis.

## Methods

### Patients

Preserved (Mankin score ≤ 4^17^) human articular cartilage samples were obtained from the femoral condyles removed during total knee replacement for osteoarthritis following informed consent (4 male and 2 female patients aged between 51 and 76). All procedures were approved by the East London and The City Research Ethics Committee 3 (ethics approval REC N. 07/Q0605/29). Full thickness explants were prepared for histology, and chondrocytes were isolated and expanded in-vitro as previously described^12^. Normal cartilage samples were retrieved from 3 patients (one 58 years-old, and two males aged respectively 14 and 16) undergone lower limb amputation because of malignant bone tumours. The samples were obtained upon favourable opinion of the Ethics Committee of the Ärztekammer Schleswig-Holstein, Bad Segeberg, Germany. We performed all procedures in accordance to relevant guidelines and regulations.

### Primary cells, cell lines and tissues

Bovine chondrocytes were isolated from metatarsal joints obtained from a local abattoir within 24 h from death. Experiments were performed when primary chondrocytes reached 80% confluence (passage 0, P0) as previously described^18^. C3H/10T1/2 cells (ATCC) were cultured according to the manufacturer’s instructions.

Murine femoral heads and patellae were explanted from 1 month-old male 129/Sv mice and processed for gene expression analysis (see below).

If not differently indicated, cells were stimulated in complete medium (DMEM/F-12 1:1 plus GlutaMax, 10% FBS, 1 mM sodium pyruvate, and 2% antibiotic antimycotic solution ThermoFisher Scientific) supplemented with human recombinant WNT3A (100 ng/ml; R&D), DKK1 (100 ng/ml, R&D), IL1B (20 ng/ml; InvivoGen), KN93 or its inactive control KN92 (10 μM; EMD Millipore), Autocamtide-2 related inhibitory peptide II, AIP, 5 μM; Calbiochem) or respective vehicles as indicated.

### siRNA preparation and transfection

Small interfering RNA for mouse CAMKIIγ was prepared using the Silencer siRNA Construction Kit (Life Technologies) following manufacturer’s instructions. siCaMKIIγ S: AAGGGTTTAAGGGTTTCACTACCTCGTGTCCTGTCTC; AS: AAACACGAAAACACGAGGTAGTGAAACCCCCTGTCTC; scramble siRNA, S: AATTCCGCAATTCCGCTCTGGACTGTAGTCCTGTCTC; AS: AAGAACTTAAGAACTTGATCAACCAGATGCCTGTCTC. The siRNA were used at 20 nM and were transfected in C3H/10T1/2 cells using JetPRIME transfection reagent (Polyplus) as described in^18^.

### RNA isolation and quantitative real time PCR

RNA isolation and PCR were performed as previously described^12^. For a complete list of primers and conditions refers to Supplementary Table 1.

### Microarray analysis

Total RNA was quantified using a Nanodrop ND-1000 Spectrophotometer and assessed for quality (QC) using the Agilent 2100 Bioanalyzer Pico kit. Five nanograms of total RNA with RNA integrity numbers (RINs) higher than 9 were selected for the microarray study. A Poly-A Spiking Control (Life Technologies) was used as amplification control.

Single primer isothermal amplification (SPIA) technology was used to generate cDNA using NuGen Ovation Pico WTA System V2 kit, following manufacturer's instructions. The SPIA cDNA was subjected to QC, fragmented and biotin-labelled using the NuGen Encore Biotin Module according to the manufacturer's instructions. The processed cDNA was subjected to a further round of QC to assess fragmentation size (Agilent 2100 Bioanalyzer Nano kit; fragment size < 200 nt).

Hybridization cocktails were prepared according to Nugen's recommendations for Human Gene 2.0 ST arrays and hybridization took place at 45 °C for 16–20 h in an Affymetrix Genechip Hybridization oven 645.

The arrays were washed and stained using wash protocol FS450_0002 as recommended by Affymetrix on the GeneChip Fluidics station 450. The arrays were scanned using the Affymetrix GeneChip Scanner. CEL files were QC checked using the Expression Console software package (Affymetrix, Thermo Fisher) by using standard metrics and guidelines for the Affymetrix microarray system. Data were normalised together using the Robust Multi-array Average (RMA) sketch algorithm.

The processing of all sample files was performed using R Programming Language (R). In brief, raw data were inputted using the 'read.celfiles' function of the 'oligo' R package^19^, with data then undergoing Robust Multiarray Average (RMA) normalisation using the 'rma' function. For cross-sample comparisons, the study design model was first created by identifying sample groupings based on KN92, KN92-WNT3a, and KN93-WNT3a status.

Raw and normalized microarray data were submitted to the Gene Expression Omnibus database at the National Center for Biotechnology Information (accession number GSE162076).

### Immunohistochemical analysis

CaMKII isoforms and phosphoCaMKII (pCaMKII) were detected by indirect immunofluorescence as described in^12^. For a list of retrieval methods, antibodies and dilutions please refer to Supplementary Table 2. All images were acquired using identical settings on an Olympus BX61 microscope and consistently modified for best rendering using Adobe Photoshop. The fluorescent and dapi images of the same field were superimposed, or the fluorescent and Nomarski images of the same field were inverted and superimposed (subtraction).

### Instability-induced OA in mice

All animal procedures were approved by the UK Home Office (PPL 70-7986). All the experimental procedures were performed in accordance to relevant guidelines and regulations. The study was carried out in compliance with ARRIVE guidelines. Mice were kept in an approved animal care facility, were housed 4 or 5 per cage in standard cages and fed ad libitum. Ten week old, male C57BL/6 mice (Charles River) were subjected to resection of the medial collateral ligament and of the anterior horn of the medial meniscus (menisco-ligament injury—MLI) as previously described^20^. In each cage, the animals were randomized to receive either PBS or KN93 (see Supplementary Table 3). The contralateral knee underwent sham surgery (arthrotomy but no damage to ligaments or menisci). The joint capsule was sutured with Vicryl 6-0 and the skin with Ethylon 5-0 sutures with atraumatic needles (Ethicon).

### Pharmacological blockade of CaMKII

Four weeks after surgery the mice were randomized to receive either the water-soluble version of the CaMKII inhibitor KN93 or PBS as control (15 animals per group). For the first 3 days KN93 (10 µmol/kg/day) or PBS were administered by intraperitoneal injection. Subsequently, ALZET osmotic minipumps (Charles River Laboratories) were inserted subcutaneously on the back of the mice allowing a continuous release of KN93 (5 µmol/kg/day) or PBS for additional 28 days. The pumps were changed once during this time. The animals were then killed by cervical dislocation.

### Histology and OA scoring

Knee joints were dissected, the majority of the soft tissue was removed, and the joints were fixed in 70% ethanol following which they were decalcified in formic acid, embedded in paraffin and sectioned as described in^7^. The sections were stained with Safranin O (SO) (0.1%, pH 4). Images were taken using the same settings on an Olympus BX61 microscope and consistently modified for best rendering using GIMP software. The extent of cartilage degradation was scored by two blinded, independent investigators using the Osteoarthritis Research Society International (OARSI) scoring system^21^. At least 5 sections/knee were scored.

### Histomorphometry

Proteoglycan loss, osteophyte size and differentiation, and subchondral bone thickness were measured by histomorphometry using ImageJ^22^. The mean values from at least 3, but on average 5 sections from each knee (1 section every 50 µm, spanning the entire depth of the joint) were calculated and used for statistical analysis.

Proteoglycan loss was analysed by densitometry of the articular cartilage as previously described^7^. In brief, images were rotated so the cartilage-bone junction in the middle of the plateau was horizontal. This is important so that non‐cartilage staining in the bone marrow spaces can be eliminated. The tibial plateau was selected to include the growth plate, any osteophytes and up to the end of the cartilage; and selections from every section were placed on one canvas, so they were perfectly aligned horizontally. To isolate the metachromatic Safranin O staining from the background and most of the orthochromatic staining (subchondral bone), the RGB image was transformed into an HSB stack. On the ‘Saturation’ slice, a rectangle 600 microns wide and the full height of the canvas was placed over the first section to include the tibial plateau, making sure to exclude any osteophytes and the “bulgy” part near the intercondylar notch. Ctrl + 1 was pressed, and then the rectangle was moved laterally to the next section and Ctrl + 2 was pressed. This was repeated until all sections were selected and then Ctrl + 3 plotted the density profile of each section. The first peak is the articular cartilage. A horizontal line was drawn to cut residual background if any, and a lateral line to limit the articular cartilage peak and eliminate any staining of bone marrow spaces. Using the wand tool to select the inside of the density profile returned the area. These results were copied from the measurements box to a statistics programme for further analysis.

Osteophytes consistently developed only on the medial tibial compartment of the operated joints. On individual sections, osteophyte size was determined by selecting the osteophyte with the polygon tool, and ensuring a global scale had been set, measuring the area. Osteophyte differentiation was assessed by comparing the intensity of SO staining with the staining of the growth plate, which was set as the threshold for comparison in Image J and defined as the staining for fully differentiated cartilage. The percentage of the thresholded area in each individual section was then normalised for the area of the osteophyte. The points in the graph represent the average of the measurements for multiple sections (at least 3/mouse).

Subchondral bone thickness was determined by selecting the subchondral area underneath the load-bearing area of the articular cartilage of the tibiae as described by Botter et al.^23^.

### Cell density

The number of cells was counted within a defined region of the articular cartilage of the tibia and the femur of the operated joint of 5 mice (1 slide/mouse) either treated with PBS or KN93. Cell density was measured as number of cells/mm^2^.

### Micro-CT scanning

Tibiae were fixed in 70% ethanol and scanned with Skyscan 1174 Micro-CT scanner (Skyscan, Antwerp, Belgium) at 53 kV, 785 µA, 0.7° rotation and 12.57 µM isometric voxel resolution. The individual slices were reconstructed using NRecon software (Skyscan) with an algorithm that included maximum ring artefact correction, 70% beam-hardening correction and misalignment compensation.

### Micro-CT analysis, Tibiae epiphyseal volume measurement

The reconstructed scan was rotated and resliced using ImageJ^22^ in a coronal orientation. Using CTAn software (Skyscan), the tibial epiphysis was reconstructed as a volume of interest (VOI) by manually selecting the epiphysis on every 10th slice for the entire stack of images. A threshold of 90 density units was set to distinguish mineralised from non-mineralised tissue within the trabeculae.

### Statistical analysis

Parametric data were compared with the t-test or Anova with Tukey-Post test for multiple comparisons. When possible, data transformation was applied to satisfy the assumptions of parametric tests as described^24^. Non-parametric data were analysed using the Wilcoxon–Mann–Whitney test. p values < 0.05 were considered significant: *p < 0.05; **p < 0.01; ***p < 0.001. For the analysis of the microarray experiment, individual comparisons within the study design model were conducted by fitting a linear model independently for each probe, with group as the *y* variable, using 'lmfit' ('limma' R package)^25^. The linear fit for each comparison was subsequently modified using the empirical Bayes ('eBayes') approach, which aims to bring the probe-wise variances across samples to common values, resulting in modified t-statistics, F-statistic, and log odds differential expression ratios. Finally, for each comparison, log_2_ fold-change (logFC), P value, and corrected P value (false discovery rate, FDR) was output. A cut-off of 10% FDR was used. Volcano plots were generated using ‘EnhancedVolcano’ (‘EnhancedVolcano’ R package)^26^.

## Results

### Transcriptional signature of the WNT3A/CaMKII pathway in the articular chondrocytes

**Figure 1:**
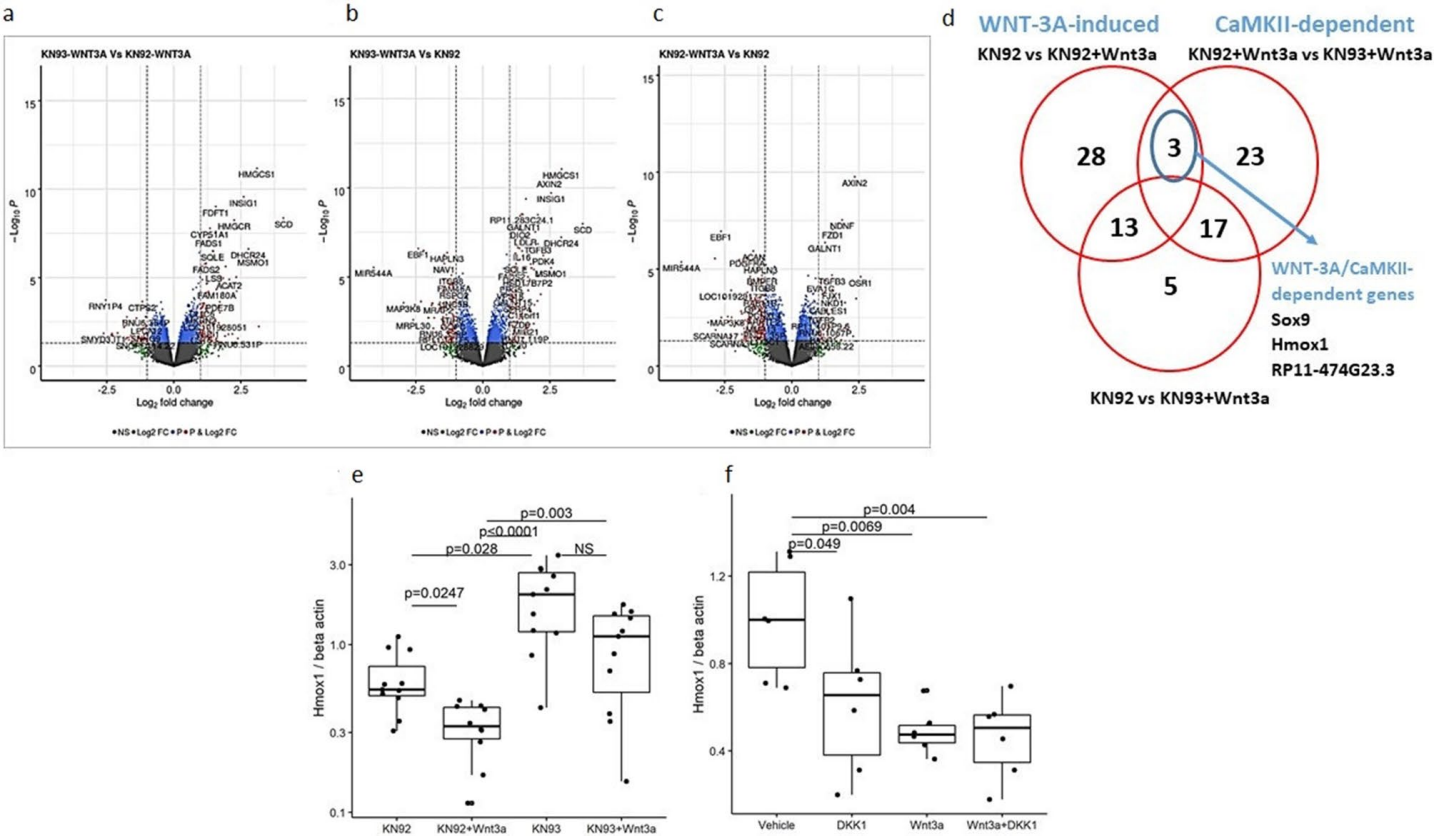
*HMOX1* is a selective target of the WNT/CaMKII pathway in the articular chondrocytes. (**a**–**d**) Human articular chondrocytes isolated from OA patients were treated with human recombinant WNT3A in presence of the CaMKII inhibitor KN93 or its inactive control molecule KN92. (**a**) Volcano plot showing genes regulated by WNT3A (KN92 + WNT3A vs KN92), (**b**) genes modulated by CaMKII inhibition in the presence of WNT3A (KN93 + WNT3A vs KN92 + WNT3A) and (**c**) genes activated by WNT3A independently of CaMKII (KN93 + WNT3A vs KN92). (**d**) Venn diagram showing the number of significantly modulated genes in each group. (**e**) qPCR confirming downregulation of *HMOX1* in response to WNT3A but not in the presence of the CaMKII inhibitor KN93. Microarray data were obtained by testing samples from 4 human donors. For 2 donors all conditions were tested in two independent cartilage explants; for one donor the condition KN92 + WNT3A was tested in a single explant; for the remaining donor all the conditions were tested in a single explant per condition. (**f**) qPCR showing that *HMOX1* is a selective target of the WNT/CaMKII branch and it is not modulated via activation of the WNT/beta-catenin pathway. n = 3 human donors, 2 technical replicates per donor. (**a**–**d**) data were analysed with a linear models with 10% FDR p value correction. (**e**,**f**) Data were analysed with one-way ANOVA followed by TukeyHSD post-hoc test.

We reported that WNT3A can activate both the WNT/beta-catenin and the WNT/CaMKII pathways in articular chondrocytes with distinct, dose-dependent biological outcomes^12^. This implies that the modulation of some transcriptional targets of WNT3A will be due to the activation of the WNT/beta-catenin-dependent pathway and others due to the activation of the CaMKII pathway. To elucidate the specific transcriptional signature of the CaMKII pathway activation by WNT3A, we performed an expression microarray analysis of human articular chondrocytes treated with WNT3A in presence of KN93 or of its inactive control compound KN92 (Fig. 1a–c). We proposed that genes that are activated in a WNT/CaMKII-dependent manner will be upregulated by WNT3A, but only in the absence of the CaMKII inhibitor KN93.

This analysis revealed that 44 genes were modulated by WNT3A in the absence of CaMKII inhibitor (Fig. 1d), but only 3 were modulated by WNT3A in a CaMKII-dependent manner (Fig. 1d). The 3 genes modulated by WNT3A in a CaMKII-dependent manner included SRY-Box Transcription Factor 9 (*SOX9)* (upregulated), the uncharacterized transcript *RP11-474G23.3* (downregulated) and Heme-Oxygenase 1 (*HMOX1*) (downregulated) mRNA. The upregulation of *SOX9* confirmed our previous results^12^. The downregulation of *HMOX1* mRNA in response to WNT/CaMKII activation was confirmed at qPCR level in human articular chondrocytes (Fig. 1e). Consistent with the requirement for CaMKII activation, KN93 effectively rescued the downregulation of *HMOX1* induced by WNT-3A and significantly upregulated its expression of this gene without exogenous WNT3A.

We previously showed that the WNT/beta-catenin pathway antagonizes the WNT/CaMKII pathway in chondrocytes and that, therefore, its suppression using DKK1 results in hyper-activation of the CaMKII-dependent targets^12^. In keeping with this model, treatment of chondrocytes with DKK1 alone decreased the expression of *HMOX1* (Fig. 1f) and did not rescue the downregulation of *HMOX1* induced by WNT3A, confirming that *HMOX1* is a negative target of the WNT3A/CaMKII pathway but not of the beta-catenin-dependent WNT pathway (Fig. 1f).

### Activated CaMKII and HMOX1 expression are inversely correlated in osteoarthritis

**Figure 2:**
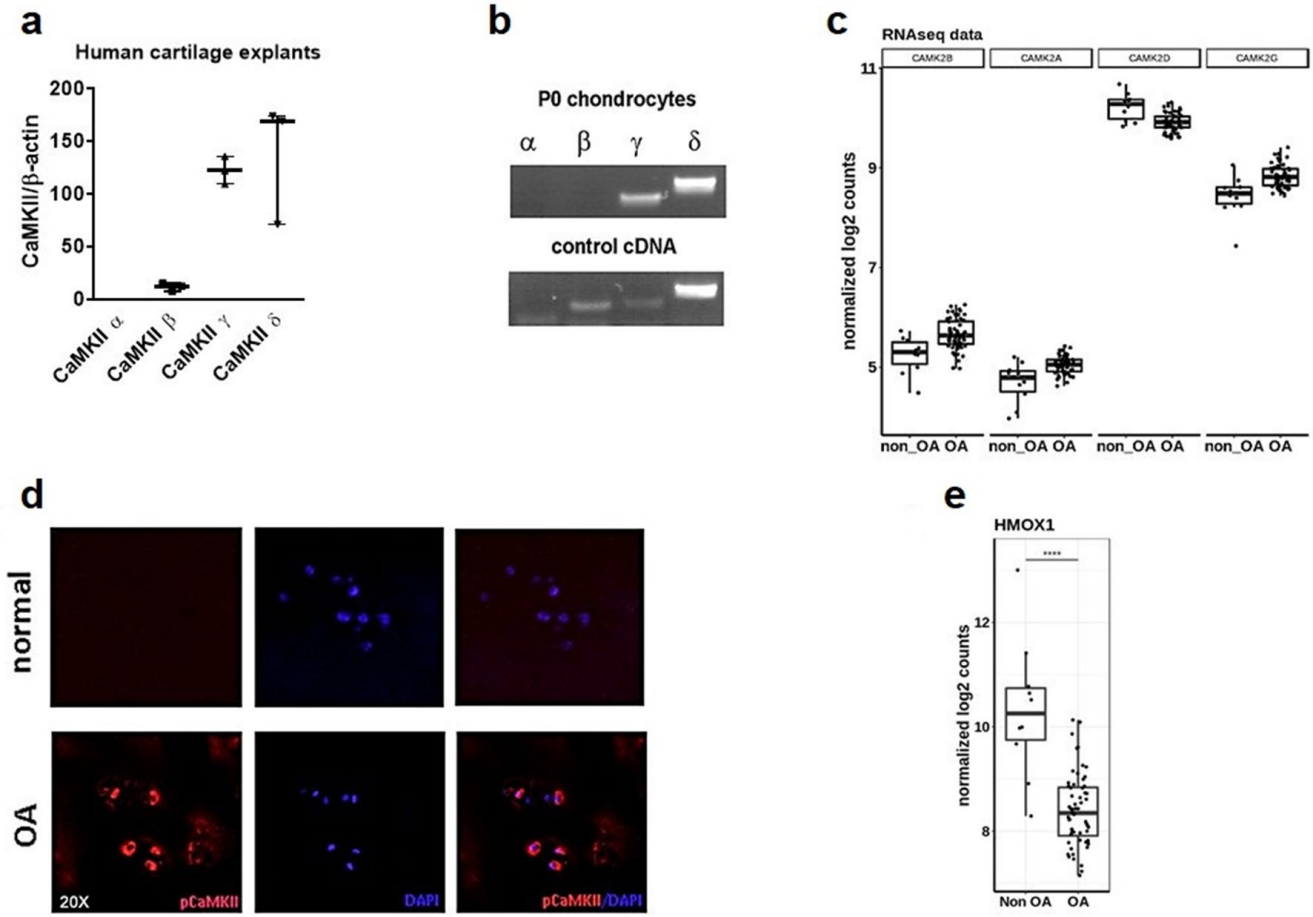
*CaMKII* and *HMOX1* expression are oppositely modulated in osteoarthritis. (**a**) Relative expression of the four CaMKII mRNA isoforms as assessed by expression microarrays in human cartilage explants^6^. (**b**) Gene expression analysis by semiquantitative PCR for *CaMKIIα, -β, -γ, -δ*, in P0 human articular chondrocytes (upper row). A pool of cDNA from brain, placenta and lymph node was used as positive control for the PCR (lower row) (n cycles = 40). All the samples were loaded and run in the same gel whose image was cut for best rendering. A picture of the full original gel can be found in Supplementary Figure 3. (**c**) RNA expression of CaMKII isoforms in human cartilage retrieved from normal and OA human cartilage as assessed by Soul and colleagues in a previously performed RNAseq^27^. (**d**) Immunodetection of phospho-CaMKII in normal and OA articular cartilage. Image representative of n = 3. (**e**) RNA deep sequencing analysis of *HMOX1* expression in normal and OA articular cartilage.

We next sought to confirm our in vitro findings in human cartilage and human OA pathology. We mined transcriptomics data in publicly available OA cartilage databases^27^. *CaMKIIγ* and *–δ* RNA were the most expressed *CaMKII* isoforms at mRNA levels in normal as well as in osteoarthritic cartilage (Fig. 2c). A similar expression pattern was detected in a previously reported expression microarray in human cartilage explants^6^ (Fig. 2a) and in cultured human chondrocytes through in vitro culture (Fig. 2b). Confirming a previous report^28^, CaMKII phosphorylation was increased in osteoarthritic cartilage as assessed with immunostaining with an antibody directed to the T287 residue (Fig. 2d). In keeping with our in vitro data showing that *HMOX1* is a negative CaMKII target, *HMOX1* expression decreased in cartilage isolated from OA patients (Fig. 2e). These results therefore suggest that the WNT/CaMKII pathway is activated in osteoarthritic cartilage. Whilst our data show a correlation between CaMKII activation and HMOX1 downregulation, they do not establish causality in vivo and other factors may contribute to HMOX1 suppression in OA.

**Figure 3:**
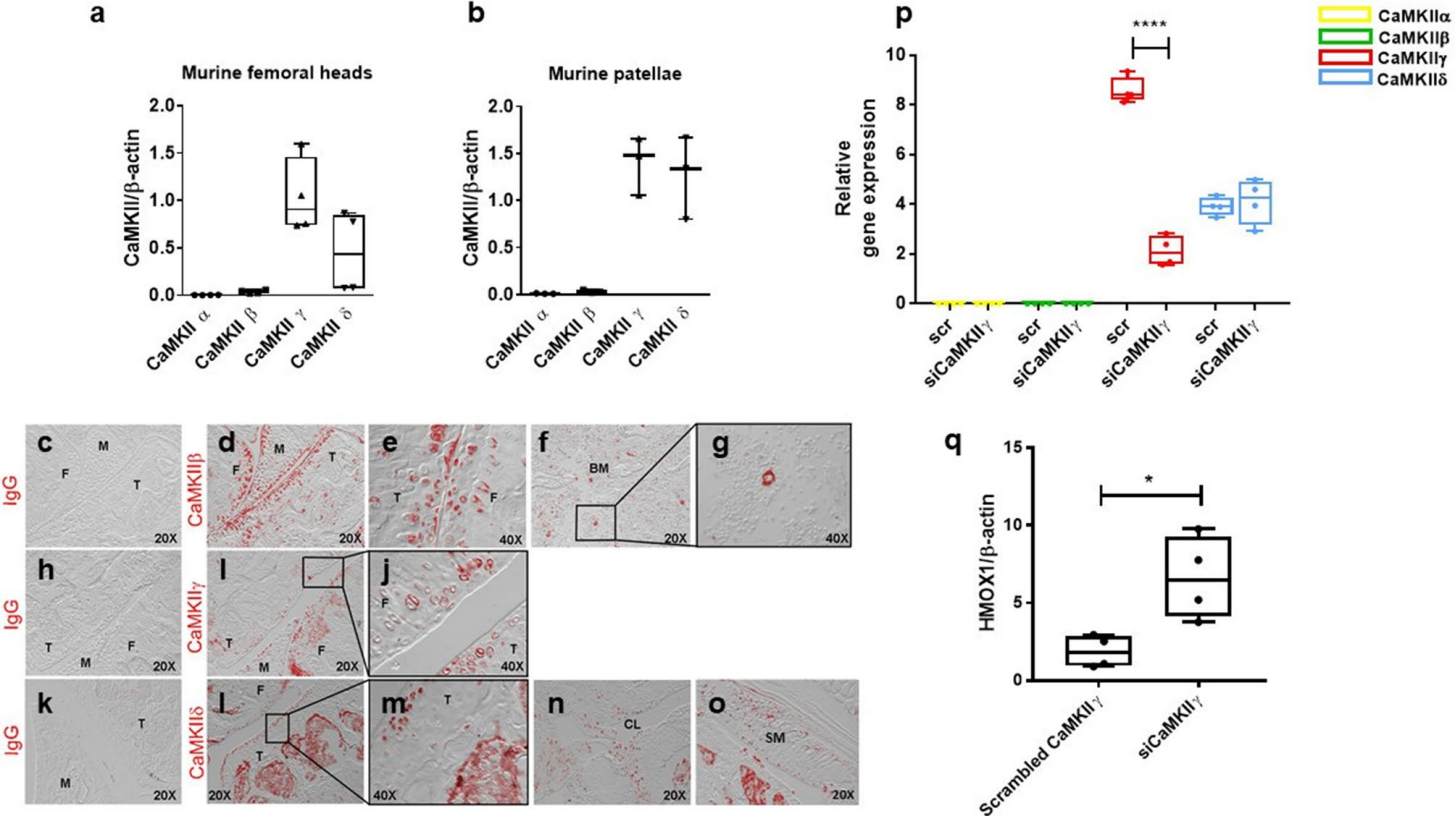
Expression of CaMKII isoforms in murine cartilage. (**a**,**b**) qPCR analysis of murine femoral heads and patellae removed from 1 month-old 129/Sv male mice (n = 4). Gene expression was normalized for the housekeeping gene beta-actin. (**c**–**o**) Immunofluorescence for CaMKII-β, -γ and δ in the murine joint. (**p**) mRNA expression of the CaMKII isoforms after CaMKIIγ silencing by siRNA in C3H/10T1/2 cells. n = 4. (**q**) Downregulation of CaMKII by siRNA was sufficient to upregulate *HMOX1* in C3H/10T1/2 cells n = 4. Data analysed by t-test. F = femur; T = tibia; M = meniscus; BM = bone marrow; CL = cruciate ligament; SM = synovial membrane.

*CaMKIIγ* and –δ were the most expressed isoforms in cartilage isolated from murine femoral heads and patellae (Fig. 3a,b), as previously seen in human cartilage. However, the different CaMKII isoforms displayed a tissue specific-expression pattern in the murine joint. CaMKIIγ was expressed in the most superficial layer of the articular cartilage, with some limited expression in the menisci (Fig. 3h–j). CaMKIIβ and –δ were also detectable in the surrounding soft tissues and in the bone marrow (Fig. 3c–g and k–o). To further validate a direct dependence of *HMOX1* downstream the activation of CaMKII, we silenced *CaMKIIγ* in the murine fibroblast cell line C3H/10T1/2 by siRNA (Fig. 3p,q). Silencing *CaMKIIγ* did not result in compensation from the other isoforms (Fig. 3p) and was sufficient to significantly upregulate *HMOX1* mRNA in these cells (Fig. 3q).

### Pharmacological inhibition of CaMKII promotes cartilage degeneration and subchondral bone remodelling in a murine model of OA

**Figure 4:**
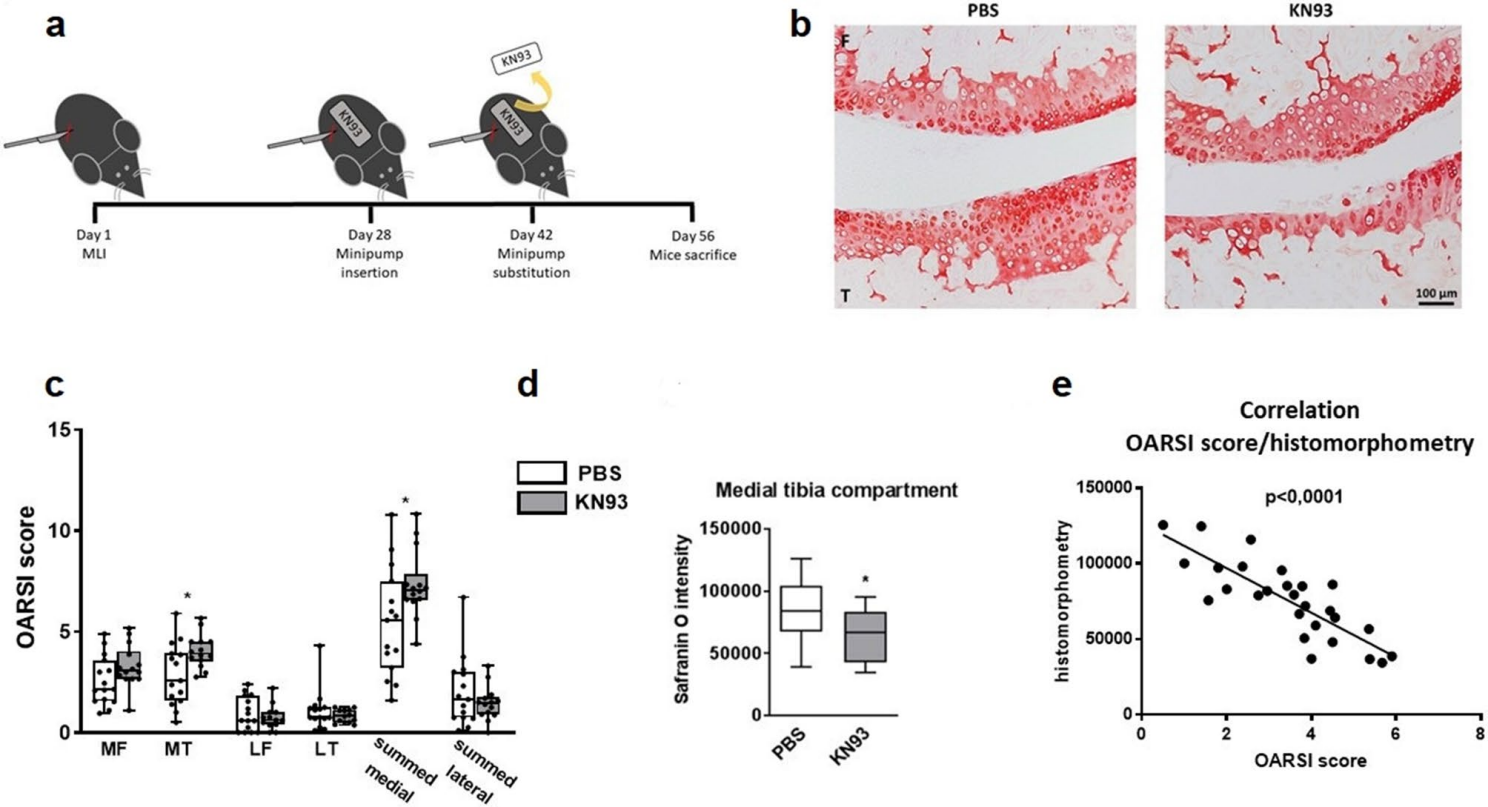
Effect of CaMKII inhibition in vivo in a murine model of OA. (**a**) Osteoarthritis was induced by removal of the anterior horn of the medial meniscus and transection of the medial collateral ligament (MLI model). Four weeks after surgery, mice were administered KN93 or PBS vehicle subcutaneously via a minipump for additional 4 weeks (the initial minipump was replaced after 2 weeks). The total volume administered over 4 weeks was equal to 400 µl. n = 15/treatment. (**b**) Safranin O staining of representative sections of the medial compartment of the knee of mice treated with PBS or KN93. (**c**) OARSI score of the medial and the lateral compartments of the MLI knees n = 15/treatment. (**d**) Histomorphometry analysis of the MLI knees. (**e**) Correlation of the OARSI score and histomorphometry analysis. MF: medial femur; MT: medial tibia; LF: lateral femur; LT: lateral tibia. OARSI scores were analysed with Mann–Whitney test with Wilcoxon post-test. Histomorphometry data were analysed by t-test. Linear regression was used to compare the OARSI scores and histomorphometry data.

To investigate if CaMKII inhibition affects the outcome of OA, we induced OA in adult mice by meniscus-ligament injury (MLI)^20,29^ and 4 weeks later we inhibited CaMKII by systemic administration of the CaMKII inhibitor KN93 or PBS as control for additional 4 weeks (Fig. 4a). The weight and general health status of the animals were monitored throughout the entire length of the stimulation and no significant difference between vehicle-treated vs inhibitor-treated animals was noted (Supplementary Table 3). CaMKII phosphorylation was effectively inhibited in KN93-treated animals (Supplementary Figure 1). Animals receiving KN93 developed more severe OA, as quantified using the OARSI scoring system (Fig. 4b,c) and a corresponding decrease in proteoglycan content (Fig. 4d,e), measured by histomorphometry. A statistically significant decrease in cell density was detected in the articular cartilage of KN93-treated animals (Supplementary Figure 2).

**Figure 5:**
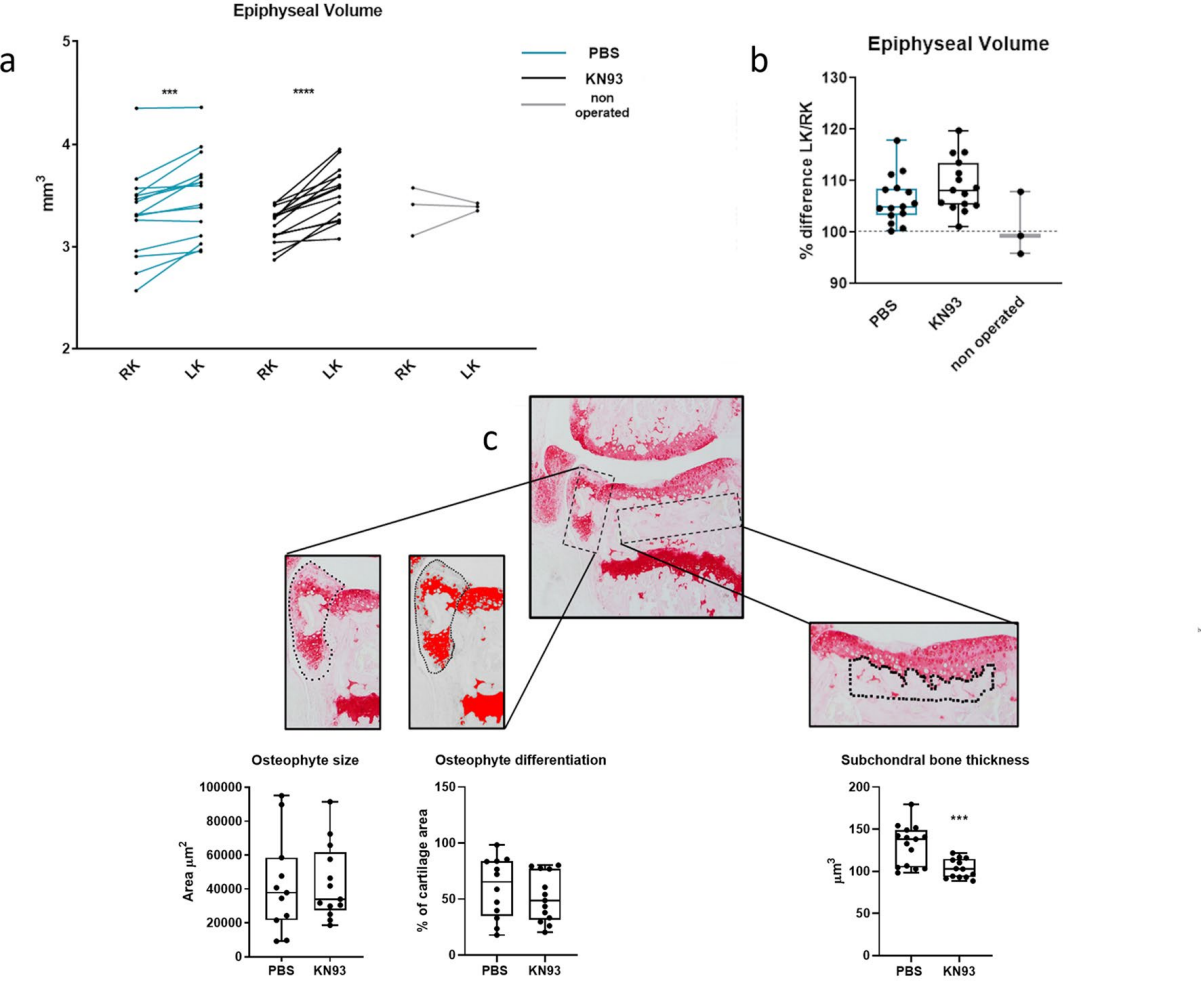
CaMKII inhibition decreases subchondral thickness. (**a**,**b**) MicroCt analysis of operated and contralateral sham-operated knee joints of mice treated with KN93 or PBS vehicle upon surgical destabilization of the knee joint. (**a**) Epiphyseal Bone volume/tissue volume of the entire tibial epiphysis of the operated knees. (**b**) Comparison of the percent change of the BV/TV between the MLI and the sham operated knee in the two treatment groups. (**c**) Histomorphometrical assessment of osteophyte area, % of cartilage area and histomorphometrical quantification of subchondral bone thickness. Data were analysed by t-test. n = 15/treatment.

Bone changes were assessed by microCt. As expected, BV/TV of the entire tibial epiphysis increased in the MLI-operated compared to the sham-operated knee, but no difference was observed between treatment groups (Fig. 5a,b). Subchondral bone thickness, however, was significantly less increased in KN93-treated mice (Fig. 5c). Osteophyte size and bone vs cartilage ratio was not affected by KN93 treatment (Fig. 5c).

### CaMKII inhibition enhanced the capacity of IL1B to upregulate catabolic enzymes in articular chondrocytes

**Figure 6:**
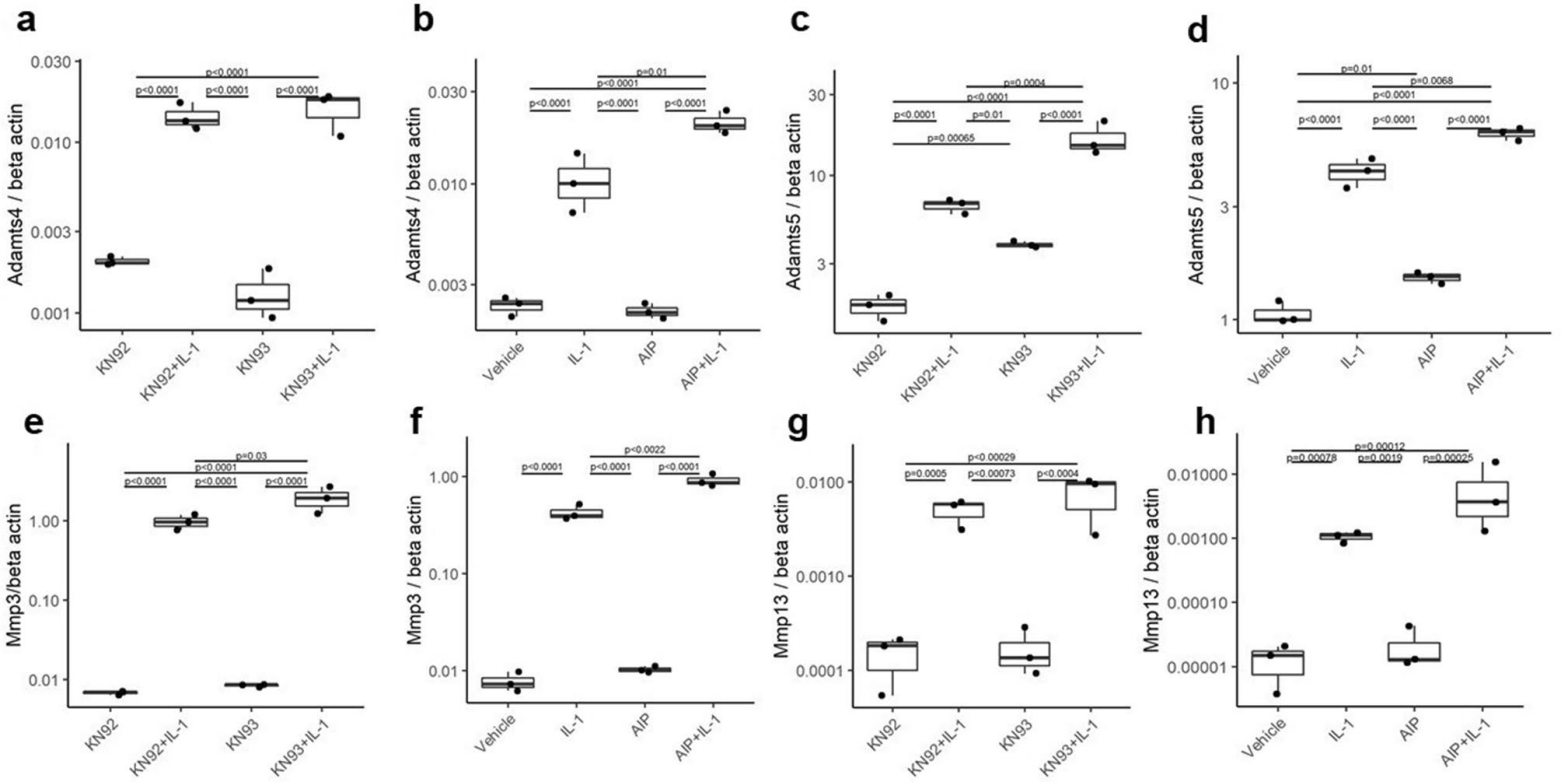
CaMKII inhibition enhanced the capacity of IL1B to upregulate catabolic enzymes in the articular chondrocytes. (**a**–**h**) mRNA expression of *ADAMTS4/5* and *MMP3/13* measured by qPCR of bovine articular chondrocytes stimulated for 24 h with IL1B (20 ng/ml) in presence or absence of KN93 or its inactive analogue KN92 (both 10 µM) (**a**,**c**,**e**,**g**) or in the presence or absence of the CaMKII inhibitor AIP (**b**,**d**,**f**,**h**) (5 µM). Data were analysed with 2 ways ANOVA and Tukey post-test. n = 3.

To gain insight into the mechanisms leading to increased cartilage degeneration in mice treated with KN93, we treated monolayer cultures of primary bovine articular chondrocytes with IL1B, a potent driver of extracellular matrix degradation^5^. CaMKII inhibition with KN93 (Fig. 6a,c,e,g) or AIP (Fig. 6b,d,f,h) induced the upregulation of the matrix-degrading enzymes MMP3 and ADAMTS5 alone and in synergy with IL1B.

## Discussion

Deregulation of Wnt signalling is associated with OA in in patients^30^ and in animal models^8,9,31^.

We previously showed that WNT3A could simultaneously activate both the beta-catenin dependent and the CaMKII-dependent pathways in chondrocytes, resulting in distinct transcriptional and biological outcomes^12^.

**Figure 7:**
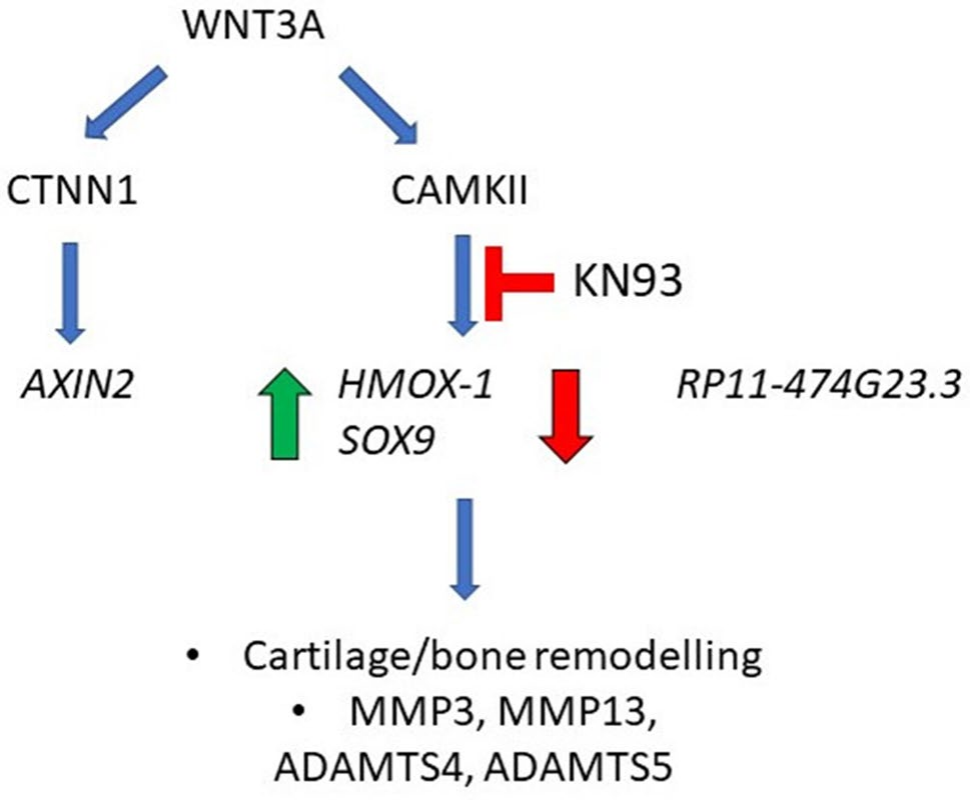
Diagram summarising the transcriptional targets modulated by WNT3A in a CaMKII dependent manner and the effect of the pharmacological blockade of CaMKII on the remodelling of joint tissues in osteoarthritis.

In this study we established the transcriptional signature of WNT/CaMKII signalling and identified *HMOX1* as a specific transcriptional target of this branch of the Wnt pathway (see schematic representation in Fig. 7). We showed that CaMKII phosphorylation was increased in human and murine OA, while the expression of *HMOX1* was decreased. CaMKII blockade increased cartilage breakdown in an instability-induced OA model in mice. CaMKII inhibition increased the capacity of IL1B to upregulate catabolic enzymes in chondrocytes.

The microarray analysis also confirmed *SOX9* mRNA upregulation in response to CaMKII blockade, as we previously showed by qPCR^12^. The mechanism by which CaMKII blockade results in *SOX9* upregulation and, simultaneously, in upregulation of cartilage-degrading enzymes is unknown. It could be speculated that *SOX9* upregulation occurs as a homeostatic response to the increased levels of extracellular matrix degradation. HMOX1 is a cytoprotective enzyme exerting antioxidant effects and chondroprotective effects in vitro^32–34^.

The three CAMKII isoforms expressed in the joint had distinct expression patterns. CaMKIIγ was selectively expressed at the articular interface in the cartilage and in the menisci, while -CaMKIIβ and CaMKIIδ had a broader expression pattern. Unfortunately, because of their high similarity across the phosphorylation site, no antibody specifically detecting the phosphorylated form of the different isoforms is available, making impossible to determine if only one isoform or all of them are activated in OA. Interestingly, however, we demonstrated that downregulation of *CaMKIIγ* alone is sufficient to mimic the effect of KN93 in inducing the upregulation of *HMOX1*, suggesting that this isoform might be downstream the activation of the WNT/CaMKII in the adult articular cartilage. The understanding of the differences in the activity of the four isoforms is going to be important for targeting CaMKII for therapeutic purposes.

In our in vivo experiments, CaMKII inhibition affected subchondral bone thickness. During embryonic development CaMKII is a modulator of chondrocyte hypertrophic differentiation during development^35,36^ and therefore we cannot exclude that altered skeletal morphogenesis may have also contributed to the worsening of the chondropathy.

CaMKII appears to modulate the final outcomes of major homeostatic effects in cartilage homeostasis in a highly context-dependent manner both embryonic development and in adulthood^12,28,35,36^. Saitta et al. also recently reported that CaMKII blockade reduced the capacity of bone morphogenetic proteins (BMPs) to upregulate cartilage phenotypic markers^37^. Calcification and PI turnover play a crucial role in OA progression^38^. It has been reported that the calcium channel TRPM7 links Ca^2+^ fluctuations to CaMKII activation and downstream signalling in growth plate chondrocytes during skeletal development^39^. It is possible that a similar mechanism may be responsible for CaMKII activation in OA articular cartilage. The identification of the calcium channels responsible for CaMKII activation in OA may offer a therapeutic opportunity. Therefore, understanding the biology of CaMKII activation is going to be key to successfully harness such pathways in musculoskeletal medicine.

## Supplementary Information

Supplementary Information.
