# Supplementary material for: Calcium calmodulin kinase II activity is required for cartilage homeostasis in osteoarthritis
Source: Sci Rep. 2021 Mar 11;11:5682. doi: 10.1038/s41598-021-82067-w (PMC7952598; doi:10.1038/s41598-021-82067-w)
Supplement: Supplementary file 1 — Supplementary Information. [file 41598_2021_82067_MOESM1_ESM.docx]

**TITLE:** Calcium calmodulin kinase II activity is required for cartilage homeostasis in osteoarthritis

**Authors:** Giovanna Nalesso, Ph.D^1^, Anne-Sophie Thorup, Ph.D^2^, Suzanne Elizabeth Eldridge, Ph.D^2^, Anna De Palma, Ph.D^1^, Amanpreet Kaur BSc^2^, Kiran Peddireddi, Ph.D^3^, Kevin Blighe, Ph.D^4^, Sharmila Rana, MRes^5^, Bryony Stott, Ph.D^6^, Tonia Louise Vincent, Ph.D, MD^6^, Bethan Lynne Thomas, Ph.D^2^, Jessica Bertrand, Ph.D^7^, Joanna Sherwood, Ph.D^8^, Antonella Fioravanti, Ph.D, MD^9^, Costantino Pitzalis, Ph.D, MD^2^, Francesco Dell’Accio, Ph.D, MD^2^.

**Affiliations:** ^1^Department of Veterinary Pre-Clinical Sciences, School of Veterinary Medicine, University of Surrey, Guildford, UK; ^2^ Barts and the London School of Medicine and Dentistry, William Harvey Research Institute, Queen Mary University of London, London, UK; ^3^MRC Clinical Trials Unit, Institute of Clinical Trials and Methodology, UCL, London UK; ^4^Clinical Bioinformatics Research, London, UK; ^5^Imperial College, London, UK; ^6^Kennedy Institute of Rheumatology, University of Oxford, Oxford, UK; ^7^Department of Orthopaedic Surgery, Otto-von-Guericke University, Magdeburg, Germany; ^8^Institute of Musculoskeletal Medicine, University Hospital Münster, Münster, Germany; ^9^Rheumatology Unit, Azienda Ospedaliera Universitaria Senese, Policlinico Le Scotte, Siena, Italy

**Corresponding authors:**

**Dr Giovanna Nalesso^1^, Ph.D**

^1^School of Veterinary Medicine, Daphne Jackson Road, GU2 7AL, Guildford, UK.

Telephone: +44 (0) 1483688676; E-mail: g.nalesso@surrey.ac.uk

**Prof Francesco Dell’Accio**

^2^William Harvey Research Institute, Charterhouse Square, EC1M 6BQ, London UK

Telephone: +44(0) 7557028104; E-mail: [f.dellaccio@qmul.ac.uk](mailto:f.dellaccio@qmul.ac.uk)

**Running head:** Role of CaMKII in osteoarthritis

**Supplementary Table 1**

| Gene | Sense | Antisense | Annealing temperature (°C) |
| --- | --- | --- | --- |
| hβ-actin | AGGAGTCGGTTGGATCGAGCA | GGGAAGGCAAAGGACTTCCTGTAAC | 55 |
| hSox9 | ACTCTGGGCAAGCTCTGGAGACT | GGCGCGGCTGGTACTTGTAGTCC | 60 |
| hHMOX1 | GAAAAGCACATCCAGGCAAT | ACTCAGGGCTTTTGGAGGTT | 55 |
| mCaMKIIα | CATTGAGGACGAAGACACCA | CCTCTGGTTCAAAGGCTGTC | 55 |
| mCaMKIIβ | TGAAGACATCGTGGCAAGAG | AGGCTTGAGGTCTCTGTGGA | 55 |
| mCaMKIIγ | CCGACGACTACCAGCTTTTC | CCTCCGGTAACAAGGTCAAA | 55 |
| mCaMKIIδ | CTGGCACACCTGGGTATCTT | ATTCTGGTGACGGAAAATCG | 55 |
| hCaMKIIα | CAGAAAAATCTGCCCAGAGC | GGTTTTGACTTGGGTTTGGA | 68 |
| hCaMKIIβ | CCTAAAGAGCAAGCCACACC | GCAGTTTCCCGAGACAGAAC | 68 |
| hCaMKIIγ | CTACCTTCAGGAGGCACGAG | AACGAAACCCTGTGGTGAAG | 60 |
| hCaMKIIδ | CCTCTGACCCTCAGTTTCCA | GGTGTCTCTCAGCCTTCTGC | 60 |
| bADAMTS4 | CCAGCTTTCCTCTCCTTGAA | GTGCAACCCACATCTGTCTG | 60 |
| bADAMTS5 | ACGGGACCGTCATGAACTAC | CTTTTGGAGCCGACTTCTTG | 55 |
| bMMP3 | TGTGTGTCTTGCCCACTAGC | TGCCTGTTGCAGAATGCTAA | 55 |
| bMMP13 | TTGAGGATTCAGGGAAGACG | TCACCAATTCCTGGGAAGAC | 60 |

List of primers and their annealing temperatures.

**Supplementary Table 2**

| Antibody | Concentration | Retrieval | Secondary AB |
| --- | --- | --- | --- |
| Anti CaMKII-β (whole antiserum) (Abcam, UK) | 1:200 | pepsin (5mg/ml in 0.02% HCl, 45’ at 37°C) +chondroitinase ABC (5mU/ml in 0.1M Tris HCl pH8, 1h at 37°C) | Cy3-conjugated goat anti-rabbit Jackson Immuno Research Laboratories, Inc. West Grove, PA, USA |
| Anti CaMKII-γ (Abcam, UK) | 5µg/µl | pepsin (5mg/ml in 0.02% HCl, 45’ at 37°C) +chondroitinase ABC (5mU/ml in 0.1M Tris HCl pH8, 1h at 37°C) | Cy3-conjugated goat anti-rabbit Jackson Immuno Research Laboratories, Inc. West Grove, PA, USA |
| Anti CaMKII-δ (Abcam, UK) | 1.25 µg/µl | citrate buffer pH6 (Dako) | Cy3-conjugated goat anti-rabbit Jackson Immuno Research Laboratories, Inc. West Grove, PA, USA |
| Anti p(Thr287) CaMKII (Cell signaling, USA) | 0.76 µg/µl | pepsin (5mg/ml in 0.02% HCl, 45’ at 37°C) | Cy3-conjugated goat anti-rabbit Jackson Immuno Research Laboratories, Inc. West Grove, PA, USA |
| Anti panCaMKII (Abcam, Cambridge, UK) | 3.5 µg/µl | pepsin (15mg/ml in 0.02% HCl, 45’ at 37°C) | Cy3-conjugated goat anti-rabbit Jackson Immuno Research Laboratories, Inc. West Grove, PA, USA |
| Anti-rabbit IgG (Abcam, UK) | Used at concentration matching the one used for the different primary antibodies | Same retrieval method used for the primary antibody | Cy3-conjugated goat anti-rabbit Jackson Immuno Research Laboratories, Inc. West Grove, PA, USA |

List of retrieval methods, antibodies used and conditions for immunostaining.

**Supplementary Table 3**

**
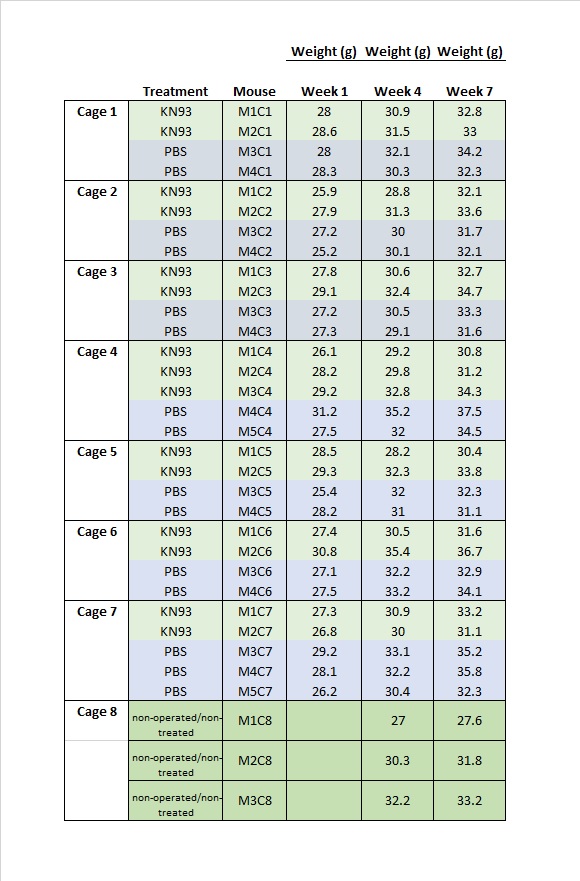
**

Treatment randomization in individual cages and weight gain throughout the experiment.

**Supplementary figure 1**

**
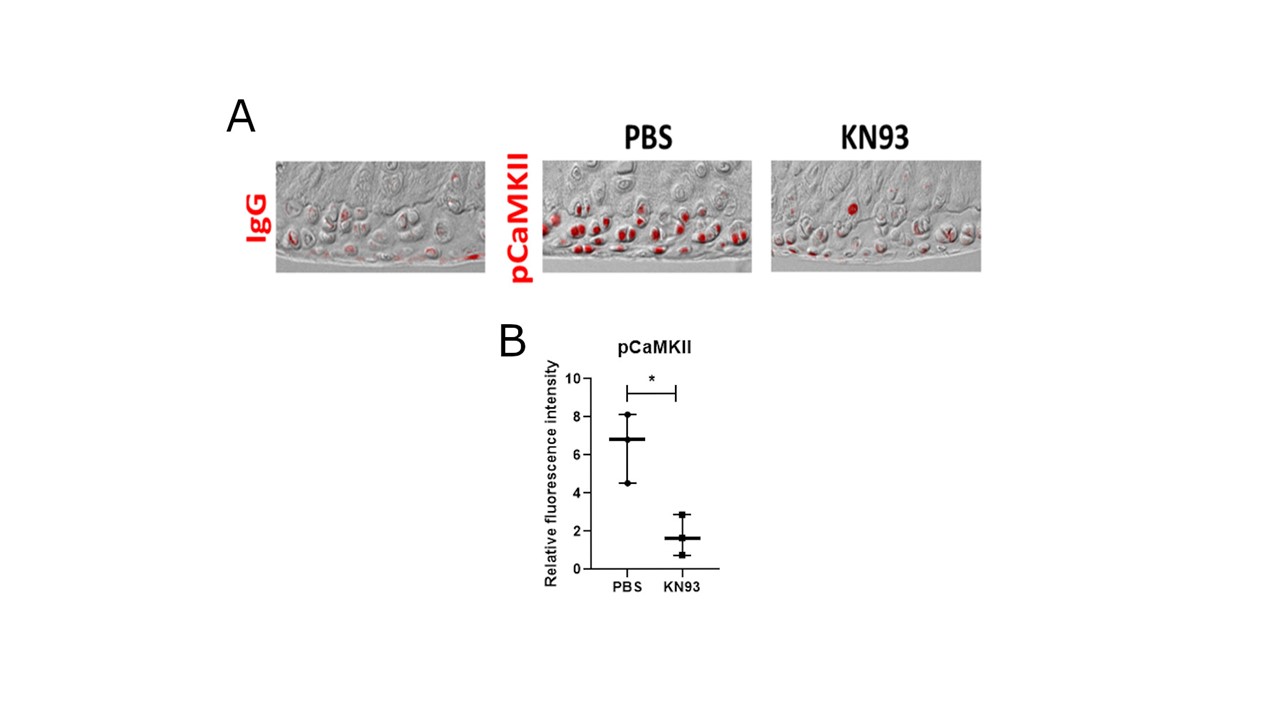
**

**Figure S1**: A) Immunofluorescence for pCaMKII in the sham operated knee of mice treated with KN93 or PBS and B) quantification of the staining. Individual data points represent the average of the pCaMKII fluorescence in the medial and lateral tibia and femur. n=3/treatment

**Supplementary Figure 2**

**
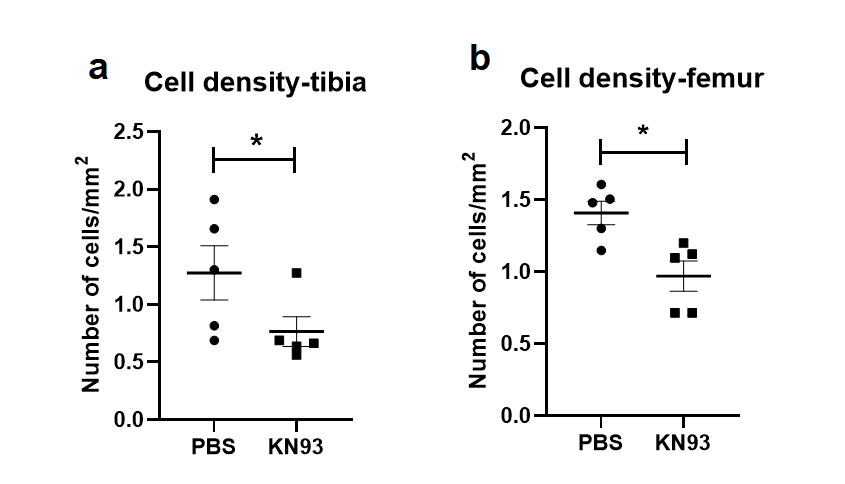
**

**Figure S2:** Number of chondrocytes per mm^2^ in the articular cartilage of operated mice. N=5; *=p<0.05

Supplementary Figure 3


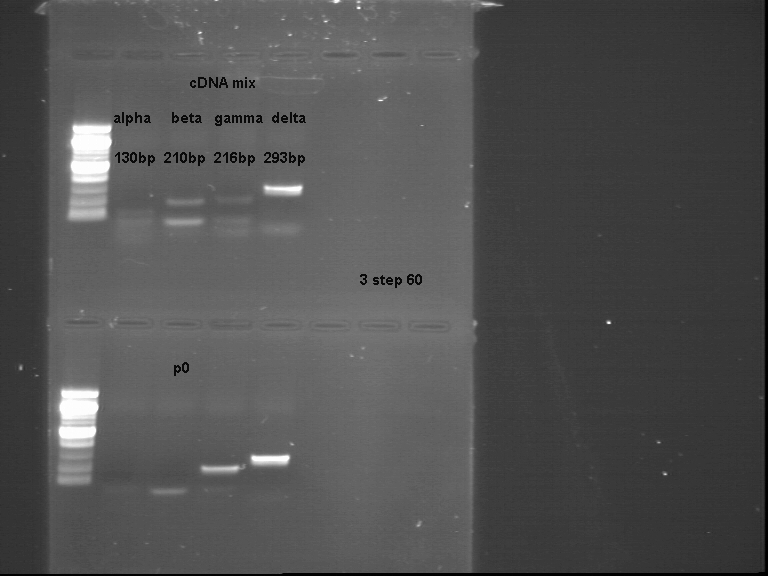


**Figure S3:** Original picture of the gel represented in Figure 2B of the main manuscript. BP= base pair, P0=passage 0 articular chondrocytes cDNA, 3 step 60= PCR settings
